# Postural Control and Functional Ankle Stability in Professional and Amateur Skateboarders

**DOI:** 10.3390/healthcare9081009

**Published:** 2021-08-06

**Authors:** Yang-Kun Ou, Zhi-Wei Chen, Chien-Nan Yeh

**Affiliations:** 1 Department of Creative Product Design, Southern Taiwan University of Science and Technology, Tainan 71005, Taiwan; ouyk@stust.edu.tw (Y.-K.O.); ma51C208@stust.edu.tw (Z.-W.C.); 2 Department of Physical Medicine and Rehabilitation, Chi-Mei Hospital, Tainan 71004, Taiwan

**Keywords:** postural control, functional ankle instability, skateboarding, behavior

## Abstract

Basic maneuvers in skateboarding, such as the ollie, put the player at high risk for ankle injuries because of the position of the feet required to perform the maneuvers. This study investigated ankle stability and reaction time for the tibialis anterior, fibularis longus, and fibularis brevis in professional and amateur skateboarders. In total, 16 professional and 16 amateur skateboarders were recruited as participants and underwent range of motion assessments, balance testing, and muscle reaction time measurements. The results revealed that professional skateboarders had a significantly smaller inversion angle compared to amateur players, which suggested better joint control and hence greater safety in the former. Balance testing results indicated better balance in professional skateboarders, and healthy skateboarders had better balance than did injured professional and amateur skateboarders. No significant difference in muscle reaction time was observed between amateur and professional skateboarders.

## 1. Introduction

Skateboarding has recently been included at the Olympic Games, representing one of five new sport programs to be introduced in the 2020 Summer Olympics in Tokyo [1]. To perform various combinations of skateboarding tricks, both ankles must be in simultaneous plantarflexion and inversion, which can cause strain on the lateral ligaments, thereby making the ligaments vulnerable to injury and the ankle joint susceptible to repeated sprains.

In general, 85% of ankle sprains involve injuries to the lateral ligaments such as, in order of increasing severity, slight or more severe swelling, tears, or massive laceration. Inadequate care of injuries may compromise the stability of the joint, which can lead to repeated sprains. Hence, the reinjury rate of lateral ligaments can reach as high as 80% [2]. Of all ankle sprains, 20% become chronically unstable, in which the ankle “gives way” and becomes more susceptible to repeated sprains [3,4].

Chronic ankle instability is most commonly believed to have two major causes: mechanical and functional ankle instability. Mechanical ankle instability is defined as ankle movement that is beyond the physiological limit of the ankle’s range of motion (ROM) due to structural damage to the ligamentous tissues that support the joint, whereas functional ankle instability is defined as the subjective feeling of ankle instability or recurrent and symptomatic ankle sprains (or both) from proprioceptive and neuromuscular deficits.

Proprioception is the perception of location, movement, and action of the parts of the body relative to each other [5]. In the case of the ankle joint, proprioception occurs from mechanoreceptors in the ligaments and joint spaces and other receptors (such as in the skin and muscles) that transmit signals regarding the movement and location of the joint to the central nervous system for coordination with reflex and neuromuscular control to achieve joint stability [6].

However, after injuries such as an ankle sprain, these receptors become damaged, which reduces the signal transmission of the proprioceptive stimuli and results in the weakening of reflex responses and joint position sense, which affects the coordination and control of the person’s posture and flexibility [6]. Deficits in functional ankle instability include balance deficits [7,8], joint position sense deficits [9], delayed peroneal muscle reaction time [10], altered common peroneal nerve function [11,12], strength deficits in the evertor muscles [13], and postural control deficits [14]. For professional athletes, ankle sprains limit movement and participation in activities, and this reduced participation in sports may affect their livelihood and cause them to incur financial loss [15]. 

Most studies on ankle injuries have focused on popular sports, such as basketball, baseball, and soccer [16,17,18]; few have investigated extreme sports, particularly skateboarding. Therefore, this study investigated ankle joint movement by comparing professional and amateur skateboarders and evaluated functional ankle instability, including through the assessment of ankle ROM, muscle reaction time, postural control, and sudden inversion.

## 2. Materials and Methods

### 2.1. Research Subjects

This study recruited 32 skateboarders aged 18–45 years who had at least 3 years of skateboarding experience. They were categorized into professional and amateur players, with 16 skateboarders in each. The professional group comprised skateboarders who had at least 3 years of skateboarding experience, had full-time work experience as professional skateboarders or as skateboarding coaches, had corporate sponsors, and could perform an 80 cm ollie and a kickflip. Skateboarders in the amateur group had at least 3 years of skateboarding experience, could perform a 50 cm ollie but not flip maneuvers, and had no titles from previous contests. All participants were required to be healthy and have normal athletic functioning, and all underwent ultrasound assessment of the knee and ankle joints by physicians from the department of rehabilitation, who assessed for and excluded individuals with previous lower limb fractures, open wounds, heart diseases, abnormal vestibular function, venous-related diseases, and pregnancy.

### 2.2. Clinical History and Investigation

This study commenced after being approved by the Institutional Review Board of Chi Mei Medical Center. Signed informed consent was obtained from each participant before the experiment began. Participants were required to fill out a questionnaire with questions on age, sex, body weight, body height, body mass index (BMI), dominant foot (the forward foot when standing on a skateboard), occupation, other leisure activities that involve the feet, past ankle injuries, past skeletal system injuries, and any neuromuscular or nervous system diseases.

For the physical examination, the participants underwent ultrasound examination at a medical center to test for diseases related to soft tissue (muscles, ligaments, and tendons) or joints as well as symptoms such as pain or discomfort in the tendons or ligaments, arthritis, and masses.

An ultrasound examination was conducted to assess each participant’s medial and lateral ligaments, bones, tendons, and muscles. During examination, the examiner first palpated the parts to be assessed to ensure the absence of pain before proceeding with the ultrasound examination. Participants in both groups were required to undergo musculoskeletal assessment with ultrasound evaluation by a physician to ensure the absence of active injuries or diseases and evidence of ankle sprains. If the examination revealed swelling or ligament laceration, then the participant was excluded from the study.

Furthermore, Romberg’s test was used to exclude sensory ataxia [19]. For Romberg’s test, participants were asked to stand with both feet together and arms by their side, first with their eyes open and then with their eyes closed for 30 s while the examiner checked for any significant swaying or loss of balance.

### 2.3. Range of Motion (ROM)

The active ROM of non-weight-bearing ankle joints was measured using a standard goniometer with the participant supine on an examination table to prevent passive loading of the ankle joint. Supination, pronation, dorsiflexion, and plantarflexion of the ankle joint were measured.

### 2.4. Muscle Reaction Time

**Figure 1:**
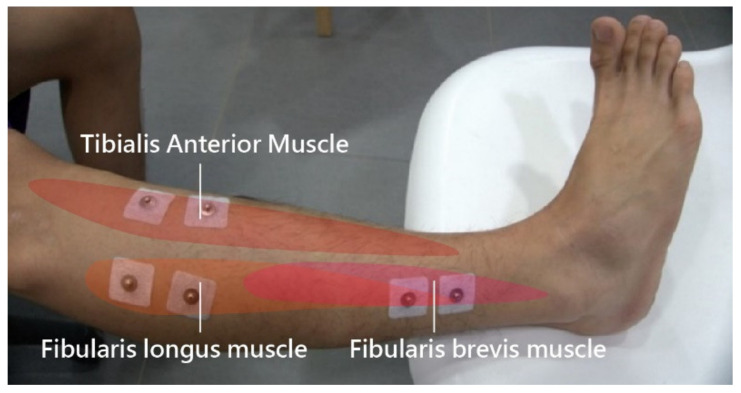
Electrodes for the wireless physiological monitor.

A BioRadio (Great Lakes NeuroTechnologies, Cleveland, Ohio) wireless physiological monitor was used in this study to simultaneously record the electromyographic (EMG) signals of three muscles. Electrodes from the wireless physiological monitor were affixed on the skin surfaces above the tibialis anterior, fibularis longus, and fibularis brevis muscles (Figure 1) on each leg. The BioRadio system was set at 24-bit resolution with sampling frequency of 1000 Hz and an input range of ±0.187 V, which was and filtered (high-pass Butterworth, 60 Hz) during acquisition. The monitor was connected to BioCapture on a computer to capture the muscle reaction time to events, such as a tilting process to simulate sudden events, including accidents. This procedure was conducted 12 times each for the dominant and non-dominant foot.

### 2.5. Postural Control

The Biodex Balance System (New York, NY, USA), a multiaxial balance device, was used in this study to assess balance and neuromuscular control, and it was connected to a touchscreen display to enable visual feedback. The foot balance platform provides a surface tilt of up to 20° to simulate sloping surfaces. The platform was tilted toward the anterior, posterior, right side, left side, anterior-right, anterior-left, posterior-right, and posterior-left. There were 12 difficulty levels for achieving postural stability, with higher levels indicating greater stability. The participants were barefoot throughout testing. 

**Figure 2:**
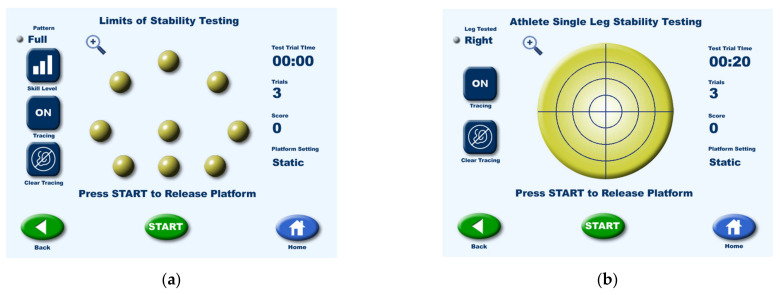
(**a**) Limits of stability testing and (**b**) Single-leg stability testing.

The limits of stability and single-leg stability were assessed separately. First, stability testing was performed at levels 12 and 8, 3 times each, with 10 s breaks in between (Figure 2a). After level 12 was completed, testing for level 8 commenced after a 10 min break. The dominant foot was tested before the non-dominant foot, with a 10 min break in between. To evaluate the single-leg stability of the dominant and non-dominant feet (Figure 2b), the participant was required to stand up straight on one leg, while the non-test leg was flexed and kept balanced at a distance from the floor and the test leg while avoiding swaying, and a target was kept at the center of the display screen. The dominant foot was tested first, and each 20 s test was performed 3 times, with 10 s breaks in between. The non-dominant foot was tested in the same fashion after a 10 min break. 

### 2.6. Tilting Platform

**Figure 3:**
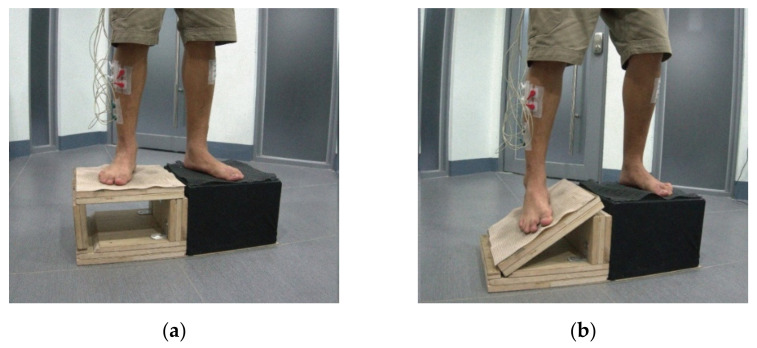
Tilting platform, shown for the right foot. (**a**) Platform at rest, and (**b**) Platform tilted at 30°.

To simulate events that lead to ankle sprain, a tilting platform made of wooden boards was constructed for this study, in which the top board (the platform surface) was supported by two boards (Figure 3a). Tilting of the platform by 30° was brought about by removing the outer supporting board (Figure 3b) for both the right and left foot.

### 2.7. Research Procedure

The study procedure consisted of 2 parts. The first part involved the collection of baseline information, conduction of Romberg’s test, and ultrasound examination of musculoskeletal structures to confirm each individual’s eligibility for participation in the study.

The second part consisted of experimental testing, in which ankle ROM, muscle reaction time, and postural stability of the ankle were evaluated. Ankle ROM data were generated, in degrees, for both the dominant and non-dominant foot during supination, pronation, dorsiflexion, and plantarflexion. Data for muscle reaction time, in seconds, were generated for tibialis anterior, fibularis longus, and fibularis brevis muscles for each leg. For postural stability, nine stability scores were generated from the device after testing: the overall score and anterior, posterior, left, right, left anterior, right anterior, left posterior, and right posterior scores. All evaluations took a total of 4 h to complete.

### 2.8. Statistical Analysis

Data were analyzed with SPSS v.22.0 statistical software (Armonk, NY: IBM Corp.). Baseline information was reported with descriptive statistics. Group comparisons of test variables were analyzed with *t*-test. The effect size was estimated using Cohen’s *d*. The level of significance used for all analyses was α < 0.05.

## 3. Results

### 3.1. Clinical History and Investigation

Table 1 presents the results of the clinical history and investigation of the subjects. Each group of professional and amateur skateboarders comprised 15 male participants and one female participant. The right and left foot were dominant in 10 and six professional skateboarders, respectively. The right and left foot were dominant in seven and nine amateur skateboarders, respectively. The average age was 25.5 (±5.5) and 20.1 (±1.6) years among professional and amateur skateboarders, respectively. The average body height was 170.8 (±7) cm and average weight was 63.1 (±10) kg among professional skateboarders. The amateur skateboarders had an average body height of 171.8 (±5.6) cm and average weight of 61.4 (±8.3) kg. The average BMI was 21.6 (±3.17) among professional skateboarders, with one individual in the overweight category, and that for amateur skateboarders was 20.8 (±2.31).

Musculoskeletal assessment revealed significant evidence of injury among professional skateboarders in the hallux (first toe), lateral ligaments, and superior portion of the tibia. All participants received treatment. Among the amateur skateboarders, four had significant evidence of sprain in the lateral ligaments, and one had an accessory navicular bone without symptoms of pain. Only one participant received treatment.

### 3.2. Ankle ROM

The means and standard deviations of ankle ROM in professional and amateur skateboarding players are listed in Table 2. Independent *t-test* analysis revealed a significant difference only in the inversion angle of the dominant foot (*t* (30) = −2.338, *p* < 0.05, *d* = 0.39). No significant difference was observed between the two groups regarding other angles of the ankle joint of the dominant and non-dominant feet.

### 3.3. Muscle Reaction Time

#### 3.3.1. Muscle Reaction Time in Professional versus Amateur Skateboarders

The means and standard deviations of muscle reaction time in professional and amateur skateboarding players are listed in Table 3. Independent *t-test* analysis revealed no significant difference between the two groups of skateboarders.

#### 3.3.2. Muscle Reaction Time in Healthy versus Injured Skateboarders

The means and standard deviations of muscle reaction time for professional and amateur skateboarding players are listed in Table 4. A comparison between healthy and injured professional skateboarders revealed no significant difference in the reaction time of the tibialis anterior, fibularis longus, or fibularis brevis.

### 3.4. Position Sense Test

#### 3.4.1. Comparison of Stability Scores between Professional and Amateur Skateboarders at Levels 12 and 8

The means and standard deviations of stability scores for professional and amateur skateboarding players at levels 12 and 8 are listed in Table 5.

Independent *t-test* analysis revealed that at level 12, professional skateboarders had higher stability scores for overall (*t*(30) = 2.224, *p* < 0.05, *d* = 0.38), right anterior (*t*(30) = 2.062, *p* < 0.05, *d* = 0.35), and right posterior (26.19 (±5.7) vs. 21.38 (±6.4), *t*(30) = 2.24, *p* < 0.05, *d* = 0.38) than amateur skateboarders. This suggested that the professional skateboarders had better postural stability. However, at level 8, no statistically significant difference in stability scores was observed between the two groups.

While comparing between levels 12 and 8, among professional skateboarders, a significant difference was observed in the left posterior stability score (*t*(30) = −3.006, *p* < 0.05, *d* = 0.48) between levels 12 and 8. On the other hand, among amateur skateboarders, a significant difference in scores between levels 12 and 8 was observed for right anterior (*t*(30) = −2.067, *p* < 0.05, *d* = 0.35) and right posterior (*t*(30) = −2.309, *p* < 0.05, *d* = 0.3).

#### 3.4.2. Comparing Stability Scores between Healthy and Injured Skateboarders at Levels 12 and 8

The means and standard deviations of stability scores for healthy and injured skateboarding players at levels 12 and 8 are listed in Table 6. There was no significant difference in stability scores between healthy and injured skateboarding players.

## 4. Discussion

As the prominence of skateboarding as a sport has increased, so has the skill level and maneuver complexity required of skateboarders. However, skateboarding maneuvers often require feet positions at high risk for ankle injuries, and the risk is even more heightened by the fact that at a peak velocity of 40 mph [20] and without any mechanical braking system in the skateboard [21], the potential force of impact in skateboarding is the greatest among all sport activities, and this creates a risk of acute and chronic injuries. Previous skateboarding studies have emphasized the description of skateboarding maneuver postures, the mechanical designs of skateboards, injury epidemiology [20,22,23], skateboarding environment, and skateboarding ecology—namely, its effects on individuals and society [24,25], but few studies have investigated skateboarders’ feet by using standardized measurements. All test participants in the present study had sustained ankle injuries from skateboarding akin to those sustained by snowboarders, gymnasts, and ballet dancers in their respective sports [26]. All of the professional skateboarders sought treatment at the time the injuries occurred, whereas only four of the amateur skateboarders did so, which indicated a greater attention to personal health among professional skateboarders than among amateur skateboarders. This conflicts with the findings of Determan [26], in which skateboarders in both categories mainly dealt with injuries through self-treatment. Regarding sprain location and musculoskeletal problems, injuries among professional skateboarders occurred more often in the anterior talofibular ligament, hallux, and knees and manifested as bruises and fractures. Amateur skateboarders all reported previous sprains in the anterior talofibular ligament, but none manifested as bruises. Although ultrasound examination revealed prominent signs of sprains among the professional skateboarders, one of whom even sustained a rupture, none complained of pain, and all exhibited normal activity during the interview and experiment. This evidence of sprain in most skateboarders agrees with the study results of Rodríguez-Rivadulla et al. [27], and reinjury often occurs after sprains. Repetitive injuries may lead to functional ankle instability, postural control deficits, and delayed peroneal muscle reaction time. Training exercises that increase proprioception are recommended for the treatment of such injuries [27].

Regarding ankle joint ROM, this study considered larger angles of foot inversion during the ollie maneuver to be unnecessary because the inversion angle of the dominant foot in professional skateboarders was significantly smaller than that of amateur skateboarders. A similar study on ballet dancers reported that the plantar flexion angle of professional dancers was larger than that of amateur dancers and healthy participants [28]. When professional dancers dance on pointe, to achieve stability, the ankle joint must be plantar flexed perpendicular against the floor, even to the point of over-plantar flexion. Therefore, the ankle plantar flexion angle in professional dancers is larger, which is contrary to the findings in this study. In our study, a review of medical history revealed that amateur skateboarders are less inclined to seek medical treatment for injuries such as lateral ligament sprains, which are one of the most common skateboarding injuries, and this may lead to ligament laxity and tendon elongation with subsequent increases in inversion angle of the dominant foot with no significant changes in the non-dominant foot. This suggests that when professional skateboarders perform an ollie, flips, or other advanced maneuvers, larger inversion angles are not necessarily better and that the ability to safely and accurately control the maneuver is more crucial. Skateboarders, when practicing ollies or flips, tend to repeatedly rub the surface of their shoe against the board while their ankles are inverted to become accustomed to an inverted posture. However, repetitive ankle inversion and forceful rubbing against the skateboard can easily cause laxity in the lateral ligaments, thereby increasing the inversion angle in amateur skateboarders.

Regarding muscle reaction time, most studies have employed an electrical tilting platform connected to an electromyography instrument to ensure a narrow range of error, and these instruments are often more advanced. By contrast, the equipment used in this study differs from that used in other studies, and muscles such as the tibialis anterior were tested, in addition to the commonly used fibularis longus and fibularis brevis. Doctors recommend testing the tibialis anterior because it is a muscle that controls the ankle joint angle. In our study, no difference in muscle reaction time was observed between professional and amateur skateboarders, which is consistent with the findings of other studies comparing the muscle reaction times of the fibularis longus and fibularis brevis between professional and amateur ballet dancers [28].

An estimated 74% of ankle sprains lead to ankle instability, which causes changes in proprioception, neuromuscular functions, and dynamic and static posture control [29]. Regarding postural stability, during testing at level 12, professional skateboarders achieved significantly higher overall, right anterior, and right posterior scores than did amateur skateboarders. However, at level 8, no significant difference was observed between professional and amateur skateboarders. Although most participants felt that stability was more difficult to achieve at level 8, professional skateboarders exhibited a significant difference in left posterior stability scores between the two levels, whereas amateur skateboarders exhibited a significant difference in the right anterior and right posterior stability scores between the two levels. Since achieving stability on the foot balance platform at level 12 was more difficult, better muscle control was required to maintain balance. This study revealed that long-term training in skateboarding improves balance more than it improves muscle control. Studies have shown that deficits in balance increase the risk of ankle injury, and proprioception training has been reported to improve balance [27]; however, in this experiment, the testing procedure did not have enough repetition for sufficient learning and training. Thus, no significant difference in balance was detected. When healthy and injured skateboarders were compared at level 12 and 8, professional skateboarders exhibited a significant difference in left posterior scores, which is consistent with the findings of previous studies that have noted that professional skateboarders with old injuries have worse postural control than do healthy ones. Similarly, healthy amateur skateboarders achieved significantly higher right anterior and right posterior scores than injured amateur skateboarders.

During failed maneuver attempts, many participants used their wrists and elbows to break falls so that their bodies would roll rather than receive direct impact. However, this may cause impact to the shoulder region. Moreover, the upper extremities are more prone to injuries than are the lower extremities [24,30]. This may explain the lack of popularity of skateboarding as a sport [31]. Ankle injuries often lead to muscle and tendon ruptures, which are accompanied by damage to mechanoreceptors. This can lead to deficits in proprioception and balance control and even affect individuals’ health in severe cases.

## 5. Conclusions

The muscle control ability of professional skateboarders was better than that of amateur skateboarders, as shown in the former having higher stability scores than the latter. The findings of this study can provide skateboarders with a better understanding of the sport so that they can adopt appropriate measures to prevent injuries and suggest that proprioception training should be employed to improve ankle joint stability. Future studies should include other body parts, such as the knees, shoulders, wrists, and elbows, in musculoskeletal ultrasound assessments and compare healthy participants with those who do not play sports involving the lower extremities.

## Figures and Tables

**Table 1:** Baseline information and medical history.

	Professional Skateboarding Players	Amateur Skateboarding Players
Age (y) Mean (SD) Range	25.5 (±5.5) 19–36	20.1 (±1.6) 18–23
Sex (*n* (%)) Male Female	15 (93.75) 1 (6.25)	15 (93.75) 1 (6.25)
Body weight (kg) Mean (SD) Range	63.1 (±10) 48–85	61.4 (±8.3) 50–80
Body height (cm) Mean (SD) Range	170.8 (±7) 159–181	171.8 (±5.6) 157–179
BMI (body mass index) Mean (SD) Range	21.6 (±3.17) 17.6–30.12	20.8 (±2.31) 17–24.97
Dominant foot (*n* (%)) Left foot Right foot	6 (37.5) 10 (62.5)	9 (56.25) 7 (43.75)
Leisure Activities involving (*n* (%))	2 (12.5)	3 (18.75)
Ankle sprain (*n* (%))	9 (56.25)	4 (25)
Treatment (*n* (%))	9 (100)	1 (25)

**Table 2:** Ankle ROM of professional and amateur skateboarding players.

		Professional Skateboarding Players	Amateur Skateboarding Players
Supination	Dominant Foot *	32.50° (±4.00°)	37.25° (±7.08°)
	Non-Dominant Foot	35.25° (±6.14°)	35.31° (±8.19°)
Pronation	Dominant Foot	23.56° (±6.32°)	24.19° (±10.08°)
	Non-Dominant Foot	26.69° (±9.30°)	21.44° (±7.32°)
Dorsiflexion	Dominant Foot	43.50° (±7.47°)	43.44° (±7.52°)
	Non-Dominant Foot	42.00° (±7.75°)	43.13° (±8.99°)
Plantarflexion	Dominant Foot	27.44° (±7.13°)	31.50° (±7.70°)
	Non-Dominant Foot	28.00° (±6.94°)	27.31° (±5.49°)

* *p* < 0.05.

**Table 3:** Muscle reaction time in professional and amateur skateboarding players.

		Professional Skateboarding Players	Amateur Skateboarding Players
Tibialis Anterior Muslce	Dominant Foot	0.3045 s (±0.04 s)	0.3078 s (±0.10 s)
	Non-Dominant Foot	0.2864 s (±0.04 s)	0.2799 s (±0.04 s)
Fibularis Longus Muscle	Dominant Foot	0.2962 s (±0.04 s)	0.2899 s (±0.03 s)
	Non-Dominant Foot	0.2793 s (±0.04 s)	0.2810 s (±0.03 s)
Fibularis Brevis Muscle	Dominant Foot	0.2786 s (±0.03 s)	0.2934 s (±0.04 s)
	Non-Dominant Foot	0.2747 s (±0.04 s)	0.2737 s (±0.03 s)

**Table 4:** Muscle reaction time in healthy and injured skateboarding players.

		Healthy Skateboarding Players	Injured Skateboarding Players
Tibialis Anterior Muslce	Dominant Foot	0.2927 s (±0.03 s)	0.3137 s (±0.05 s)
	Non-Dominant Foot	0.2727 s (±0.04 s)	0.2894 s (±0.03 s)
Fibularis Longus Muscle	Dominant Foot	0.2858 s (±0.02 s)	0.3043 s (±0.05 s)
	Non-Dominant Foot	0.2645 s (±0.04 s)	0.2832 s (±0.03 s)
Fibularis Brevis Muscle	Dominant Foot	0.2749 s (±0.04 s)	0.2814 s (±0.02 s)
	Non-Dominant Foot	0.2555 s (±0.04 s)	0.2828 s (±0.03 s)

**Table 5:** Stability scores in professional and amateur skateboarding players.

		Professional Skateboarding Players	Amateur Skateboarding Players
Level 12	Overall	25.25 (±4.30)	21.88 (±4.29)
	Anterior	29.94 (±5.92)	29.25 (±11.96)
	Posterior	29.31 (±6.72)	28.00 (±13.65)
	Left	32.50 (±9.19)	27.31 (±9.80)
	Right	30.75 (±7.00)	26.25 (±6.70)
	Left Anterior	28.13 (±7.77)	28.31 (±8.63)
	Right Anterior	30.38 (±8.04)	24.44 (±8.25)
	Left Posterior	24.88 (±7.21)	25.63 (±11.00)
	Right Posterior	26.19 (±5.72)	21.38 (±6.42)
Level 8	Overall	26.44 (±4.79)	24.19 (±4.45)
	Anterior	32.19 (±9.47)	29.19 (±7.51)
	Posterior	32.31 (±12.76)	32.81 (±15.65)
	Left	28.13 (±8.05)	25.81 (±8.08)
	Right	29.56 (±8.49)	26.38 (±6.11)
	Left Anterior	33.88 (±9.75)	32.25 (±8.08)
	Right Anterior	33.31 (±8.77)	30.75 (±9.01)
	Left Posterior	33.31 (±8.61)	27.88 (±7.59)
	Right Posterior	27.75 (±7.13)	26.31 (±5.65)

**Table 6:** Stability scores in healthy and injured skateboarding players.

		Healthy Skateboarding Players	Injured Skateboarding Players
Level 12	Overall	23.33 (±3.63)	24.67 (±3.52)
	Anterior	31.00 (±13.01)	30.50 (±7.06)
	Posterior	29.17 (±14.86)	32.83 (±15.87)
	Left	27.58 (±10.06)	28.08 (±6.99)
	Right	28.17 (±6.41)	26.83 (±4.82)
	Left Anterior	31.17 (±7.69)	33.75 (±8.13)
	Right Anterior	26.92 (±7.74)	30.92 (±7.97)
	Left Posterior	28.17 (±11.09)	28.08 (±6.46)
	Right Posterior	23.58 (±5.25)	26.00 (±5.70)
Level 8	Overall	17.50 (±3.11)	22.75 (±7.04)
	Anterior	24.00 (±6.78)	25.25 (±8.46)
	Posterior	24.50 (±9.98)	32.75 (±17.35)
	Left	26.50 (±10.41)	19.00 (±8.04)
	Right	20.50 (±3.87)	25.00 (±9.90)
	Left Anterior	19.75 (±5.06)	27.75 (±6.95)
	Right Anterior	17.00 (±4.69)	30.25 (±13.15)
	Left Posterior	18.00 (±7.07)	27.24 (±11.59)
	Right Posterior	14.75 (±5.19)	27.25 (±6.24)

## Data Availability

The data presented in this study are available on request from the corresponding author. The data are not publicly available due to legal restrictions imposed by the government of Taiwan in relation to the “Personal Information Protection Act”.
